# Somatosensory Evoked Potentials and Central Motor Conduction Times in children with dystonia and their correlation with outcomes from Deep Brain Stimulation of the Globus pallidus internus

**DOI:** 10.1016/j.clinph.2017.11.017

**Published:** 2018-02

**Authors:** Verity M. McClelland, Doreen Fialho, Denise Flexney-Briscoe, Graham E. Holder, Markus C. Elze, Hortensia Gimeno, Ata Siddiqui, Kerry Mills, Richard Selway, Jean-Pierre Lin

**Affiliations:** a Department of Basic and Clinical Neuroscience, Maurice Wohl Clinical Neuroscience Institute, Institute of Psychiatry, Psychology and Neuroscience, King’s College London, London SE5 9RX, United Kingdom; b Complex Motor Disorder Service, Children’s Neurosciences Department, Evelina Children’s Hospital, Guy’s and St Thomas’ NHS Foundation Trust, London SE1 7EH, United Kingdom; c Department of Clinical Neurophysiology, King’s College Hospital NHS Foundation Trust, Denmark Hill, London SE5 9RS, United Kingdom; d Moorfields Eye Hospital NHS Foundation Trust, 162 City Road, London EC1V 2PD, United Kingdom; e University College London Institute of Ophthalmology, 11-43 Bath St, London EC1V 9EL, United Kingdom; f F. Hoffmann-La Roche AG, Biostatistics, 4070 Basel, Switzerland; g Department of Psychology, Institute of Psychiatry, Psychology and Neuroscience, King’s College London, United Kingdom; h Department of Radiology, Guy’s and St. Thomas’ NHS Foundation Trust, London, United Kingdom; i Department of Neuroradiology King’s College Hospital NHS Foundation Trust, London, United Kingdom; j Department of Functional Neurosurgery, King’s College Hospital NHS Foundation Trust, Denmark Hill, London SE5 9RS, United Kingdom

**Keywords:** CMCT, Central Motor Conduction Time, SEP, Somatosensory Evoked Potential, DBS, Deep Brain Stimulation, CP, Cerebral Palsy, BFMDRS-m, Burke-Fahn-Marsden Dystonia Rating Scale motor score, HIE, Hypoxic Ischemic Encephalopathy, COPM-p, Canadian Occupational Performance Measure-performance, TMS, Transcranial Magnetic Stimulation, MEP, Motor Evoked Potential, MRI, Magnetic Resonance Imaging, CST, Corticospinal Tract, Dystonia, Secondary, Deep Brain Stimulation, Central Motor Conduction Time, Somatosensory Evoked Potentials, Dystonic cerebral palsy, Children

## Abstract

**Objectives:**

To report Somatosensory Evoked Potentials (SEPs) and Central Motor Conduction Times (CMCT) in children with dystonia and to test the hypothesis that these parameters predict outcome from Deep Brain Stimulation (DBS).

**Methods:**

180 children with dystonia underwent assessment for Globus pallidus internus (GPi) DBS, mean age 10 years (range 2.5–19). CMCT to each limb was calculated using Transcranial Magnetic Stimulation. Median and posterior tibial nerve SEPs were recorded over contralateral and midline centro-parietal scalp. Structural abnormalities were assessed with cranial MRI. One-year outcome from DBS was assessed as percentage improvement in Burke-Fahn-Marsden Dystonia Rating Scale (BFMDRS-m).

**Results:**

Abnormal CMCTs and SEPs were found in 19% and 47% of children respectively and were observed more frequently in secondary than primary dystonia. Of children proceeding to DBS, better outcome was seen in those with normal (*n* = 78/89) versus abnormal CMCT (*n* = 11/89) (*p* = 0.002) and those with normal (*n* = 35/51) versus abnormal SEPs (*n* = 16/51) (*p* = 0.001). These relationships were independent of dystonia aetiology and cranial MRI findings.

**Conclusions:**

CMCTs and SEPs provide objective evidence of motor and sensory pathway dysfunction in children with dystonia and relate to DBS outcome.

**Significance:**

CMCTs and SEPs can contribute to patient selection and counselling of families about potential benefit from neuromodulation for dystonia.

## Highlights

• A high proportion (47%) of children with dystonia have evidence of abnormal sensory pathway function.
• Central motor conduction times (CMCTs) and somatosensory evoked potentials (SEPs) show a significant relationship with deep brain stimulation (DBS) outcome, independent of aetiology or cranial MRI.
• CMCTs and SEPs can guide patient selection and help counsel families about potential benefit of DBS.

## 1 Introduction

The benefits of Deep Brain Stimulation (DBS) of the Globus Pallidus internus (GPi) for isolated idiopathic or genetic (formerly termed primary) dystonia are now well recognised in both adults and children, with improvements of 52–88% being seen in the Burke-Fahn-Marsden Dystonia Rating Scale motor score (BFMDRS-m) (Coubes et al., 2004, Vidailhet et al., 2005, Zorzi et al., 2005, Alterman and Snyder, 2007). However, isolated dystonia, in which the dystonic movements are the only feature of the neurological disease and structural neuroimaging is normal, is rare, especially in childhood (Roubertie et al., 2002). Much more common are the acquired (formerly termed secondary) dystonias, in which the movement disorder arises from a neurological disease processes or brain insult. These may be further divided into those arising from static lesions (e.g. following hypoxic ischemic encephalopathy (HIE), extreme prematurity, infection, a metabolic disturbance or vascular event) and those due to degenerative disorders. There are growing reports of children with acquired dystonia, such as dyskinetic cerebral palsy (CP) arising from perinatal brain injury, benefitting from DBS (Coubes et al., 2004, Eltahawy et al., 2004, Zorzi et al., 2005, Alterman and Snyder, 2007; Katsakiori et al., 2009, Vidailhet et al., 2009, Air et al., 2011, Marks et al., 2011, Gimeno et al., 2013; Koy et al., 2013, Lumsden et al., 2013b; Marks et al., 2013, Olaya et al., 2013, Romito et al., 2015). These children respond more modestly, with mean improvement in BFMDRS usually less than 25% (Coubes et al., 2004, Eltahawy et al., 2004, Zorzi et al., 2005, Alterman and Snyder, 2007; Katsakiori et al., 2009, Vidailhet et al., 2009, Marks et al., 2011, Gimeno et al., 2013; Koy et al., 2013, Lumsden et al., 2013b; Marks et al., 2013), but the benefits gained can significantly enhance the families’ daily lives through improved comfort and daily care (Gimeno et al., 2012, Gimeno et al., 2013; Olaya et al., 2013, Elze et al., 2016). Referrals of such children are increasing, but outcomes vary significantly across this widely heterogeneous population (−7 to 77% change in BFMDRS-m), even within individual studies (e.g. −7 to 55% reported by Vidailhet et al. (2009) 0–40% reported by Olaya et al. (2013)). This makes it difficult to predict the degree of benefit an individual child may gain from neuromodulation (Austin et al., 2017). Even within the isolated dystonia group some DYT1-negative children show little or no response to DBS (Gimeno et al., 2014). Appropriate family counselling is essential to manage expectations before a child undergoes functional neurosurgery, which is not without risk (Kaminska et al., 2017, Moro, 2016), but predictive markers of DBS outcomes in acquired and complex dystonias are lacking (Koy and Timmermann, 2017). Although structural imaging abnormalities on cranial MRI are generally associated with less benefit from DBS (Eltahawy et al., 2004, Romito et al., 2015), outcomes still vary widely, regardless of whether MRI is normal or abnormal (Zorzi et al., 2005, Krause et al., 2006, Vidailhet et al., 2009); imaging evidence of structural abnormality does not necessarily correlate with function (Lumsden et al., 2015). Somatosensory Evoked Potentials (SEPs) and Central Motor Conduction Times (CMCT) have been performed in all children being assessed for possible DBS within our service since 2009 and 2007 respectively, to provide objective evidence of sensory and motor pathway function. This study reports CMCT and SEP data from a cohort of 180 children with dystonia and, for those proceeding to DBS, tests the hypothesis that these neurophysiological markers are associated with outcome.

## 2 Methods

Data were reviewed from 180 consecutive children (88 female, 92 male) with medically refractory dystonia who attended King’s College Hospital between September 2007 and September 2015 for CMCT and/or SEPs as part of their assessment for possible pallidal Deep Brain Stimulation via the Complex Motor Disorders Service at Evelina London Children’s Hospital. CMCT and MRI data from 62 children were reported previously (McClelland et al., 2011). The neurophysiological studies were performed as part of a standard clinical work-up, along with detailed imaging and a multi-disciplinary clinical assessment by a paediatric neurologist, nurse specialist, physiotherapist, occupational therapist, speech and language therapist and clinical psychologist. This was a retrospective analysis of data collected via standard clinical practice and thus formal ethical approval was not required under NHS research governance arrangements.

### 2.1 Clinical assessments

The children were examined by a consultant paediatric neurologist with expertise in movement disorders (JPL). Dystonia was classified in line with previous publications (Lin et al., 2014, McClelland et al., 2016) and with the Albanese dystonia classification (Albanese et al., 2013), taking into account the clinical characteristics and aetiology (Table 1). Dystonia severity was assessed using the BFMDRS-m at baseline. DBS outcome was primarily assessed as change in dystonia severity, expressed as percentage improvement in BFMDRS-m from baseline to 1 year post-operatively (as used widely in the DBS literature). The percentage change has the disadvantage that it tends to under-estimate improvement for severely affected individuals (i.e. with higher baseline scores), and over-estimate improvement for less severely affected individuals. Therefore the absolute change in BFMDRS was also calculated. In addition, change in functional performance was assessed, using the Canadian Occupational Performance Measure-performance (COPM-P), a goal-orientated assessment tool which scores the level of performance regarding a set of skills and activities, from 1 to 10 (Gimeno et al., 2014). A two point change is considered clinically significant.

**Table 1:** Distribution of patients across different aetiological groups. Only the patients with technically satisfactory data are included.

Aetiological group New classification^b^	Aetiological group Previous classification^b^	Number (%) of patients with satisfactory SEP data^a^	Number (%) of patients with satisfactory CMCT data^a^
**Isolated idiopathic/genetic** Early onset generalised isolated dystonia	**Primary**	8 (7.8) (Of whom 5 DYT1 positive)	14 (9.6) (Of whom 7 DYT1 positive)
**Complex idiopathic/genetic** Early onset generalised dystonia with associated features (normal cranial MRI)	**Primary plus**	13 (12.7) (Of whom 3 DYT11 positive and 1 TIFF1 positive)	19 (13) (Of whom 2 DYT11 positive and 1 TIFF1 positive)
**Acquired non-degenerative** due to perinatal brain injury	**Secondary static –** dyskinetic cerebral palsy^c^	50 (49)	68 (46.6)
**Acquired non-degenerative** due to metabolic condition	**Secondary static –** metabolic	7 (6.9)	11 (7.5)
**Acquired non-degenerative** due to other cause	**Secondary static –** other cause	18 (18.6)	24 (16.4)
**Acquired Degenerative** due to Neuronal degeneration with Brain Iron Accumulation or mitochondrial	**Secondary progressive** (Neuronal degeneration with Brain Iron Accumulation or mitochondrial)	4 (4.9)	10 (6.8)

**TOTAL**		100	146

a 100/103 (97%) of children tested for SEPs had technically satisfactory data. 146/174 (84%) children tested for CMCT had technically satisfactory data.

b A definition of each group according to both the new dystonia classification (Albanese et al., 2013) and the previous classification used in literature from this group and others is provided to facilitate comparison with past and future studies.

c The term “*dyskinetic cerebral* palsy” implies CP dominated by *dystonia*, *choreo-athetosis*, or both, according to the Surveillance of Cerebral Palsy in Europe Study 2000 (Surveillance of Cerebral Palsy in E, 2000). None of the children in this cohort had pure choreo-athetosis without dystonia.

### 2.2 Neurophysiological recordings

Data were acquired with a NIHON KODON Neuropack M1 MEB9200 EMG/EP system. Electrical stimuli of 0·2 ms duration were applied at 2·1 Hz to the median nerve at the wrist or the posterior tibial nerve at the ankle, just above motor threshold. Median nerve SEPs were recorded over ipsilateral Erb's point, the 7th and 2nd cervical vertebra and contralateral centro-parietal scalp overlying sensory cortex at C3′ and C4′ (2 cm posterior to C3 and C4). Posterior tibial nerve SEPs were recorded primarily over ipsilateral popliteal fossa and midline scalp at Cz′ (2 cm posterior to Cz). Fz (mid-frontal) was the reference for upper and lower limb recordings. Due to a modification in protocol, a more recent sub-group of children also had lower limb SEPs recorded with a Ci - Cc or a Cz′ – Cc derivation (where i = ipsilateral and c = contralateral) (Miura et al., 2003). The upper lumbar component was recorded where possible (T12/L1 – iliac crest). Filter band-pass was 1–500 Hz. At least two averages of 250 artefact-free trials were recorded to determine reproducibility.

**Fig. 1:**
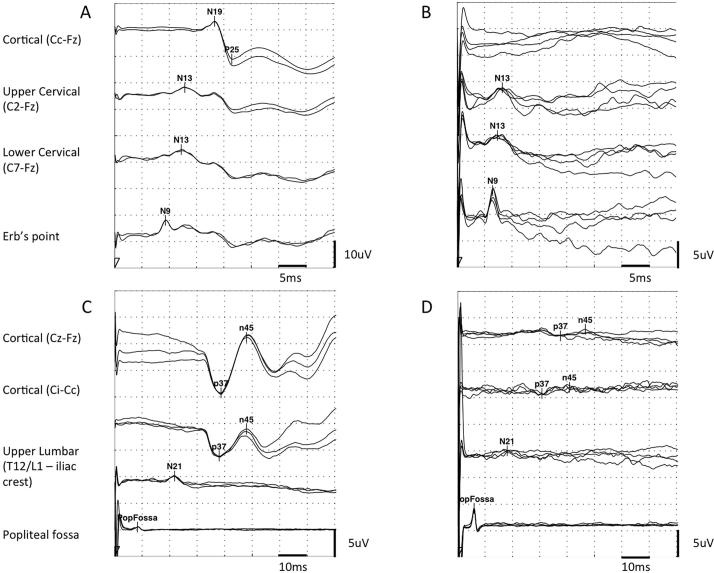
Example traces. (A) Normal upper limb SEPs from a 13 year-old child with isolated genetic (DYT1) dystonia. (B) Upper limb SEPs from a 5 year old child with Dyskinetic Cerebral Palsy secondary to term HIE showing absent cortical response despite normal cervical responses. (C) Normal lower limb SEPs from same patient as A. (D) Lower limb SEPs from a 12 year old child with Dyskinetic Cerebral palsy secondary to prematurity (25 weeks) showing low-amplitude, poorly formed cortical waveform despite adequate peripheral and spinal responses.

Analysis focused on the first negative and first positive cortical components. The components were labeled according to their polarity and peak-times in adults (equivalent to N19 and P25 for upper limb SEPs and to P37 and N45 for lower limb SEPs, see Fig. 1). Peak-times and central sensory conduction time (cortical to cervical/lumbar component inter-peak interval) were compared with published paediatric norms, taking into account age and height (Taylor and Fagan, 1988; Boor et al., 1998a, Boor et al., 1998b). Cortical potentials were classed as abnormal if they were delayed (peak times greater than published mean for age-group + 2·5 standard deviations), absent or of abnormal waveform (i.e. clear time-locked cortical activity was present but waveforms were poorly formed or broadened) (see Fig. 1). Technically unsatisfactory recordings were excluded from further analysis.

CMCT was assessed using Transcranial Magnetic Stimulation (TMS) and the F-wave method and interpreted in relation to established norms, as published previously (McClelland et al., 2011). CMCT reaches adult values by age 3 years for upper limbs (Eyre et al., 1991) and by age 6 years for lower limbs (Muller et al., 1992). A CMCT was considered abnormal if it was prolonged or the MEP to that limb was absent. For the purposes of statistical analysis, any cases in whom a prolonged CMCT was obtained which could have been physiological, due to immaturity, were excluded from the analysis (see McClelland et al., 2011 for discussion).

For each individual, there were a maximum of 4 limbs of SEP data and 4 limbs of CMCT data, but not all children had satisfactory data recorded from all 4 limbs. Results for individual limbs are therefore reported briefly for transparency, but in general the results are then simplified by considering the result for an individual child. For a given child, if CMCT to one or more limbs was abnormal, that child was considered in the “abnormal CMCT” group. Likewise, if the cortical SEP from one or more limbs was abnormal, that child was considered in the “abnormal SEP” group.

### 2.3 Imaging

**Fig. 2:**
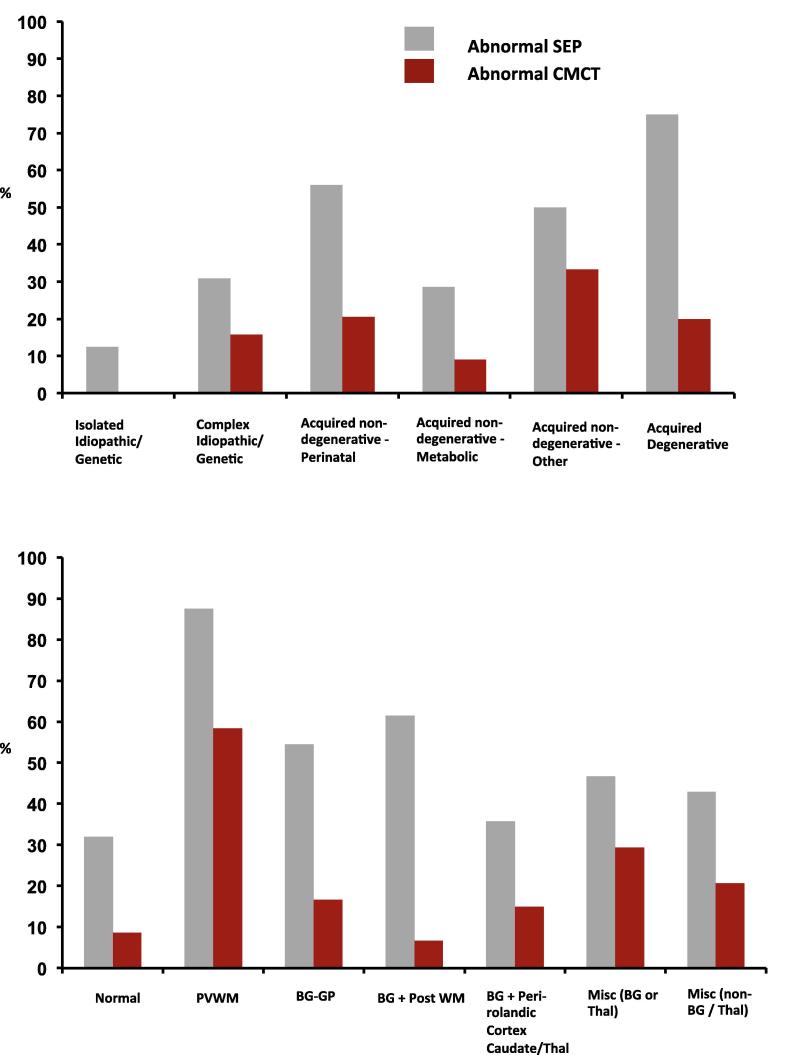
(A) Distribution of abnormal neurophysiology results across aetiological groups. Bars show percentage of children in each aetiological group with one or more abnormal cortical SEP (grey) or CMCT result (red). Note different denominators: CMCT abnormal in 0/14 Genetic/Idiopathic Isolated, 3/19 Idiopathic Complex, 14/68 Acquired perinatal CP, 1/11 Acquired metabolic, 8/24 Acquired non-degenerative “Other” and 2/10 Acquired degenerative. SEP abnormal in 1/8 Genetic/Idiopathic Isolated, 4/13 Idiopathic Complex, 28/50 Acquired perinatal CP, 2/7 Acquired metabolic, 9/18 Acquired non-degenerative “Other” and 3/4 Acquired degenerative patients. (B) Distribution of abnormal neurophysiology results across Clinical MRI Diagnostic Categories. Bars show percentage of children in each imaging group with one or more abnormal cortical SEP (grey) or CMCT result (red). Imaging abnormality in PVWM = Periventricular white matter, BG-GP = basal ganglia including globus pallidus, BG+Post WM = basal ganglia and posterior white matter. Misc. (BG or Thal) = miscellaneous minor imaging abnormalities involving thalamus or basal ganglia other than globus pallidus. Misc (non-BG/Thal) = miscellaneous minor imaging abnormalities not involving the basal ganglia or thalamus. (For interpretation of the references to colour in this figure legend, the reader is referred to the web version of this article.)

The children underwent MR examination on an Achieva 1·5 Tesla MRI system (Philips, Best, Netherlands), under general anaesthesia. Images were acquired using an 8-channel head coil, according to the local “DBS protocol”, to include those sequences required by the neurosurgeons for electrode targeting in the event that the patient went forward for DBS surgery. The sequences included are described previously (McClelland et al., 2011). Recent protocol modification includes a volumetric T1-weighted sequence with axial and coronal reformats (replacing coronal and sagittal T1-weighted) and a volumetric proton density sequence (replacing the thin axial T1-weighted and dual echo sequence for the basal ganglia). The MRI scans were interpreted by a consultant neuroradiologist and the findings classified, for the purposes of this study, on the anatomical location of abnormalities (see Fig. 2), similar to our previous publication (McClelland et al., 2011) and based on a modified version of the classifications used by Bax et al. (2006) and Robinson et al. (2009). The rationale was to identify abnormalities in the target nucleus (globus pallidus internus) and to identify those patterns of abnormality on scan which would be expected to be associated with dysfunction of the Corticospinal tract.

### 2.4 Neurosurgical procedure

For children proceeding to DBS, surgery was performed under isofluorane general anaesthesia in all patients, in view of the young age of the children. Stereotactic MRI was performed pre-operatively under anaesthesia with a Leksell G Frame in place to determine co-ordinates targeted in the postero-latero-ventral GPi. Bilateral stimulation was used in each case. For each side, prior to insertion of the stimulating electrode, single-pass microelectrode data were obtained: the presence or absence of active cells at each level assisted in identification of the GPe, medial medullary lamina and GPi, thus guiding electrode tip position (Lumsden et al., 2013a; McClelland et al., 2016). Once the target had been confirmed or amended the microelectrode was withdrawn and the stimulating electrode inserted along the same track (quadripolar DBS electrode model 3389, Medtronic, Minneapolis, MN, USA). The GPi was the target in all cases except two children in whom MRI indicated virtually complete destruction of the globus pallidus. The sub-thalamic nucleus was targeted in these two cases. Final electrode placement was confirmed by post-operative stereotactic CT scan, under the same general anaesthetic, fused with the intra-operative in-frame pre-surgical MRI. The pulse generator was then inserted (Soletra, Kinetra, Activa PC or Activa RC pulse generators, Medtronic, Minneapolis, MN, USA).

### 2.5 Statistical analysis

Prior to analysis, our key hypotheses were (1) children with abnormal SEPs show less improvement following DBS than those with normal SEPs, (2) children with abnormal CMCT show less improvement following DBS than those with normal CMCT.

Initial data analysis was masked to outcome from DBS. CMCT and SEP data for each individual were then analysed in relation to dystonia aetiology, imaging and DBS outcome. Not all children had data for both measures. For children in whom both CMCT and SEP data were available (*n* = 77), the combined results were summarized as follows: Both CMCT and SEP normal, Either CMCT or SEP abnormal, Both CMCT and SEP abnormal. This allowed evaluation of the secondary hypothesis that children in whom CMCT and SEP are both abnormal have worse outcome than those in whom one or neither test is abnormal.

Data analysis was performed in SPSS and R version 3.3.1. Differences in numeric data between groups were investigated using Kruskal-Wallis and Mann-Whitney tests for percentage change in BFMDRS (non-normally distributed data) and non-paired *t*-tests for COPM scores (approximately normally distributed data). For these three key hypotheses, the significance level was set at 0·01. To assess possible confounding, sub-group analyses were performed (using a significance level of 0·05) with regards to dystonia aetiology (acquired versus idiopathic/genetic dystonia) and imaging findings. As an additional sensitivity analysis for confounding, linear regression models were fitted in a data-driven approach (see Supplementary Material for methods).

## 3 Results

### 3.1 CMCT data

Of 176 children tested, technically satisfactory CMCT data were obtained for 427 limbs in 146 children (age range 2.5–19 years, mean 10·1, SD 4·4) (see Table 1). CMCT findings for 58/427 limbs (14%) were abnormal comprising 42 (10%) delayed responses and 12 absent MEPs (4%). Twenty-eight children (19%) had an abnormal CMCT result (prolonged CMCT or absent MEP) to at least one limb. The distribution of children with abnormal CMCTs across the aetiological groups is shown in Fig. 2A. Abnormal CMCTs were seen in a higher proportion of children with acquired (22%) than idiopathic/genetic dystonia (9%) (non-significant trend Fisher’s Exact Test *p* = 0.088). Of the idiopathic/genetic group, abnormal CMCT was seen only in *complex* idiopathic/genetic dystonia and was not seen in *isolated* idiopathic/genetic dystonia.

### 3.2 SEP data

Of 103 children tested, technically satisfactory SEP data were obtained from 368 limbs in 100 children, age range 2–19 years, mean 9·8, SD 4·4) (see Table 1). Fewer children had SEP data than CMCT because SEP testing was introduced to the assessment regimen more recently. Cortical potentials from 100/368 limbs (27%) were abnormal, comprising 47 delayed, 13 absent and 40 responses with abnormal waveform. Almost half of all children (47%) showed at least one abnormal cortical potential. The Central Sensory Conduction Time could only be calculated in a subset of patients/recordings and, where available, confirmed central delay in six responses with delayed cortical peak times. In one patient it was normal despite delay in cortical responses (see Supplementary Material for further details). The distribution of children with abnormal SEPs by aetiology is shown in Fig. 2A. One child with idiopathic isolated dystonia showed apparently prolonged cortical peak times from the lower limbs bilaterally compared with age-matched normative data (Boor et al., 1998a) but that child’s height was above the 99·6th centile for her age (148 cm; age 8 years), and the SEPs probably show no clinically significant abnormality. Abnormal SEPs were seen more often in acquired (53%) than idiopathic/genetic dystonia (24%) (Fisher’s Exact Test *p* = 0.017) and were seen most frequently in children with dyskinetic CP and in those with acquired degenerative dystonia (due to Neuronal Degeneration with Brain Iron Accumulation or mitochondrial disease).

### 3.3 Analysis by imaging sub-group

The distribution of children with abnormal CMCT and SEP in relation to imaging is shown in Fig. 2B. Abnormal CMCT and SEPs were identified in children across all imaging categories, including those with normal MRI, of whom 8/25 (32%) had abnormal SEPs and 3/35 (9%) had abnormal CMCT. Abnormal SEPs and CMCT were most frequently present in the children with evidence of periventricular white matter damage on MRI.

### 3.4 Severity data

Four children did not proceed to the full assessment and therefore did not have baseline BFMDRS-m scores measured. Severity data were available for 142 children. The majority of children’s baseline BFMDRS-m scores exceed 50. The median score was 85 (range 7–115). For the cohort as a whole, there was no significant difference in the baseline BFMDRS-m scores of those children who proceeded to DBS (*n* = 104, median score 83.0) and those who did not (*n* = 38, median score 91.3) (Mann Whitney test *p* = 0.163).

### 3.5 CMCT in relation to DBS dystonia outcome

**Fig. 3:**
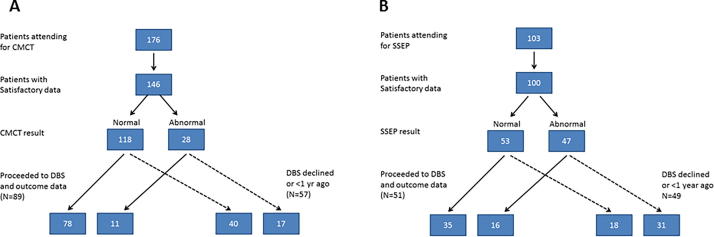
Flow charts showing numbers of children attending for each test (A = CMCT, B = SEP) and numbers proceeding to DBS.

**Fig. 4:**
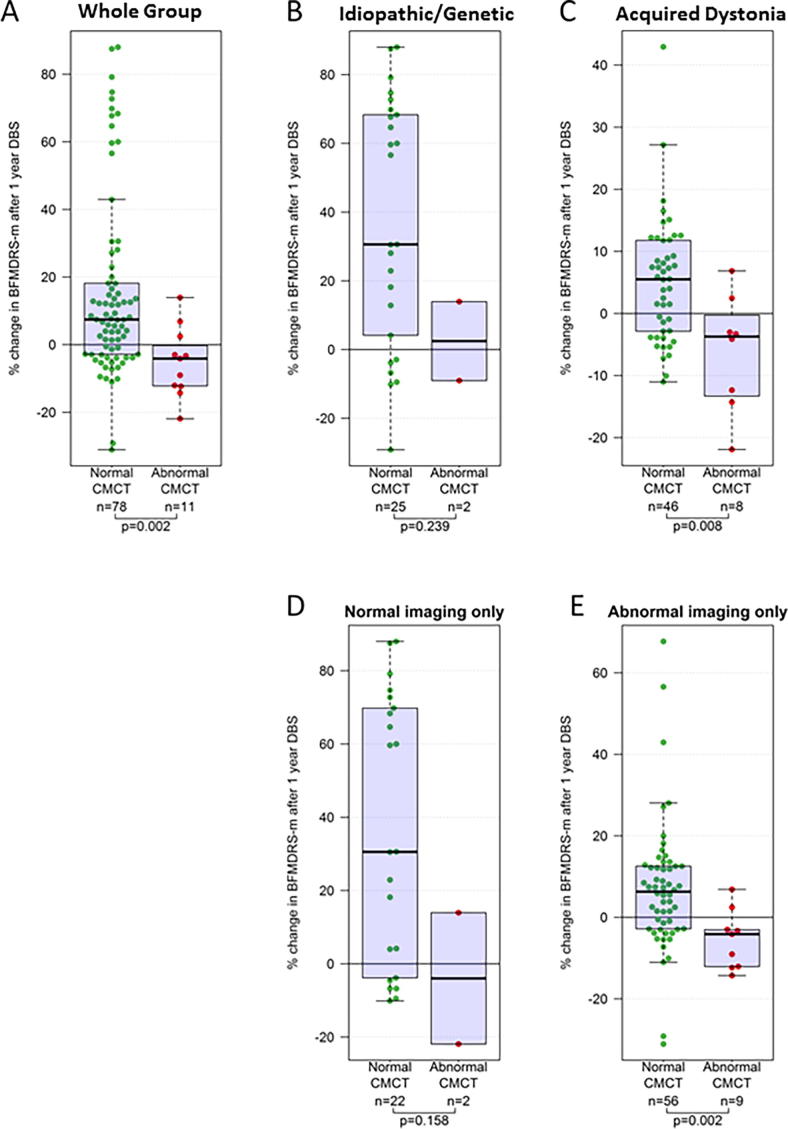
Box and Whisker plots showing outcome from DBS measured as percentage change in BFMDRS-motor score at 1 year after surgery in relation to CMCT result. (A) Results for the whole group (B–E) sub-group analyses for (B) Idiopathic/Genetic dystonia, (C) Acquired dystonia, (D) Normal cranial MRI, (E) Abnormal cranial MRI. The p-values are calculated using Mann-Whitney tests. Note different scales for C and E as improvements are generally smaller in these groups.

Eighty-nine children with CMCT data went forward for DBS and had 1 year outcome data available (see Figs. 3A, 4 and Table S1 – Supplementary Material). There was no significant difference in *baseline* severity for those with normal CMCT (*n* = 78, median score 78.3) versus those with abnormal CMCT (*n* = 11, median score 88.5) (Mann Whitney test *p* = 0.407). However, children with normal CMCT had significantly better outcomes than those with abnormal CMCT (Mann Whitney test *p* = 0.002). Sub-group analyses showed this relationship was maintained regardless of aetiology or the presence/absence of cranial MRI abnormality (Fig. 4B–E). Statistical significance was lost for some sub-groups due to the smaller sample size. Results were qualitatively similar using both percentage and absolute change in BFMDRS-m score.

### 3.6 SEP in relation to DBS dystonia outcome

**Fig. 5:**
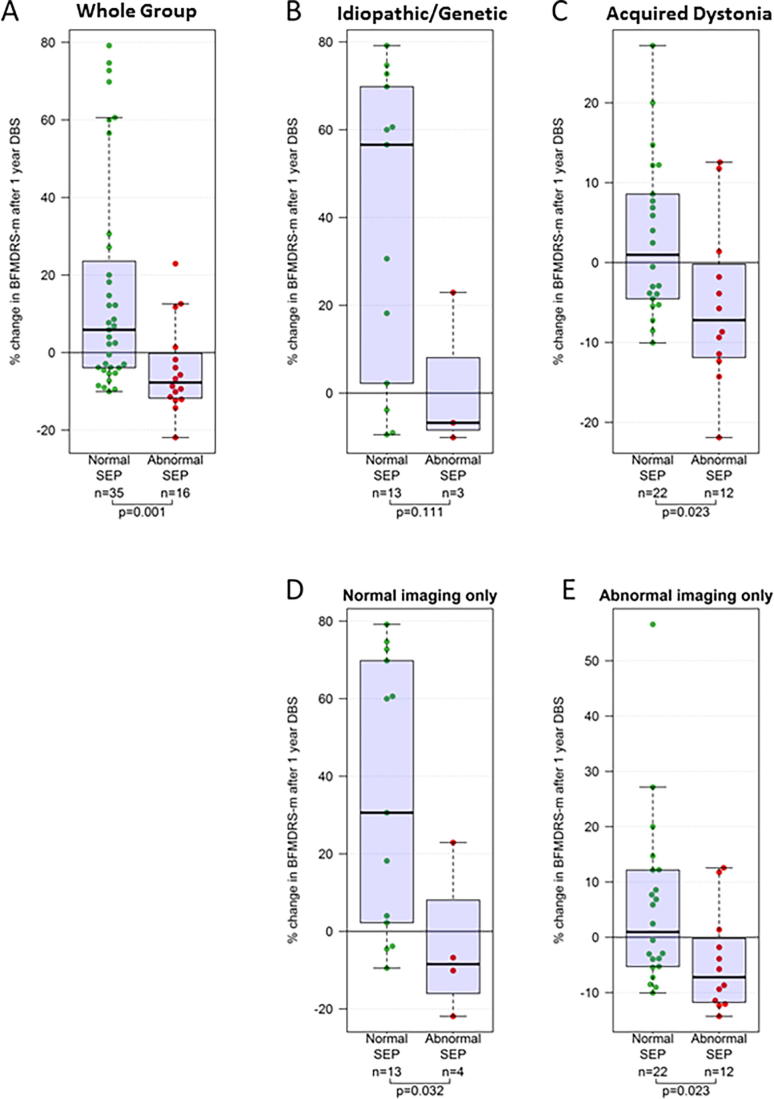
Box and Whisker plots showing outcome from DBS measured as percentage change in BFMDRS-motor score at 1 year after surgery in relation to SEP result. (A) Results for the whole group (B–E) sub-group analyses for (B) Idiopathic/genetic dystonia, (C) Acquired dystonia, (D) Normal cranial MRI, (E) Abnormal cranial MRI. The p-values are calculated using Mann-Whitney tests. Note different scales for C and E as improvements are generally smaller in these groups.

Fifty-one children with SEP data proceeded to DBS and had 1 year outcome data available (Figs. 3B, 5 and Table S1 – Supplementary Material). There was no significant difference in *baseline* severity for those with normal SEP (*n* = 35, median score 74.0) versus those with abnormal SEP (*n* = 16, median score 86) (Mann Whitney test *p* = 0.094). However, children with normal SEP had significantly higher outcome scores than those with abnormal SEP (Mann Whitney test *p* = 0.001). Sub-group analyses showed this relationship was maintained regardless of aetiology or the presence/absence of cranial MRI abnormality (Fig. 5B–E). Statistical significance was lost for some sub-groups due to the smaller sample size. Again, results were qualitatively similar using both percentage and absolute change in BFMDRS-m score.

### 3.7 Combined neurophysiology results in relation to DBS dystonia outcome

**Fig. 6:**
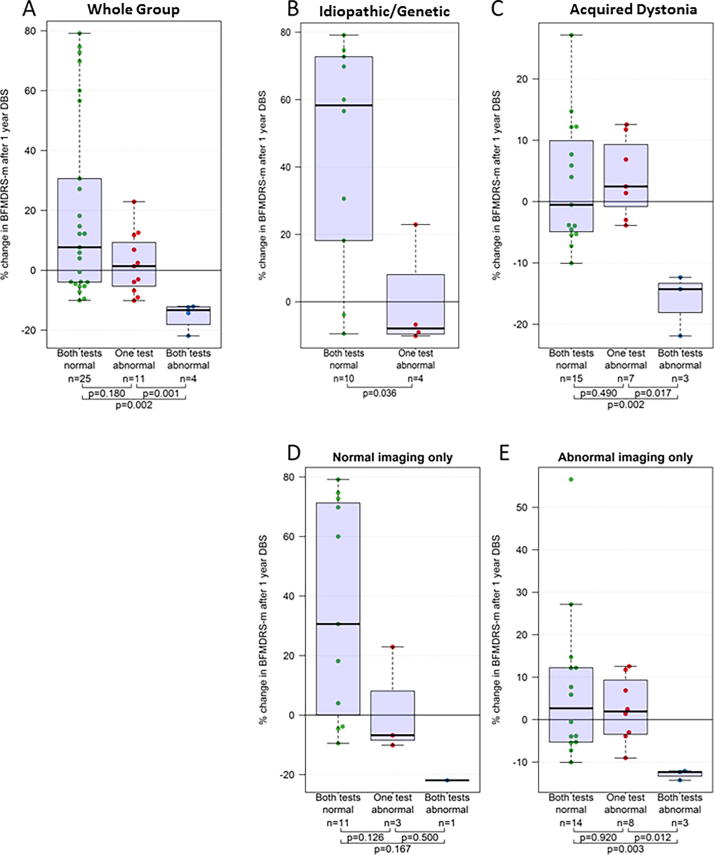
Box and Whisker plots showing outcome from DBS measured as percentage change in BFMDRS-motor score at 1 year after surgery in relation to Neurophysiology score. (A) Results for the whole group (B–E) sub-group analyses for (B) Idiopathic/Genetic dystonia, (C) Acquired dystonia, (D) Normal cranial MRI, (E) Abnormal cranial MRI. Kruskal Wallis test shows significant difference across groups for A (*p* = 0.002), C (*p* = 0.018) and E (*p* = 0.022). The p-values shown on the figures are calculated using Mann-Whitney tests. Note different scales for C and E as improvements are generally smaller in these groups.

Outcome data were available for 40 children with both CMCT and SEP data (Fig. 6, Table S2 – Supplementary Material). Kruskal-Wallis test showed a significant effect of the Combined Neurophysiology results on outcome (*p* = 0.002). For the four children in whom both CMCT and SEP were abnormal, BFMDRS-m scores deteriorated substantially following DBS. Post-hoc Mann Whitney tests showed significantly worse outcome for the “both tests abnormal” group compared with the “both tests normal” group (*p* = 0.002) and significantly worse outcome for the “both tests abnormal” group versus the “one test abnormal” group (*p* = 0.001). Sub-group analyses again showed that this relationship was maintained regardless of aetiology or the presence/absence of cranial MRI abnormality (Fig. 6B–E). Statistical significance was lost for some sub-groups due to the smaller sample size. Again, results were qualitatively similar using both percentage and absolute change in BFMDRS-m score.

### 3.8 Outcomes for children receiving STN-DBS

Two children received STN rather than GPi stimulation, because their MRI indicated virtually complete destruction of the globus pallidus. One had bilateral pallidal necrosis following a febrile illness. This child had both normal CMCT and normal SEP and showed a 27% BFMDRS-m improvement at 1 year. The second child had methylmalonic acidaemia with MRI showing cystic changes to both Globus pallidi. This child had normal CMCT but did not have SEPs recorded (having attended our service prior to our including SEP in the assessment). They did not show any improvement in BFMDRS-m at 1 year (% change of −2.8%). Inclusion/exclusion of their data from the analysis did not make any significant change to the overall results.

### 3.9 Multiple regression analysis

As an additional check for potential confounding, linear models were fitted with variables chosen in a data-driven approach. The % change in BFMDRS-m score was modelled in relation to dystonia aetiology (acquired versus idiopathic/genetic), cranial MRI (normal vs abnormal), CMCT result (normal vs abnormal), and SEP result (normal vs. abnormal). Two models were identified that showed similar predictive performance (Table 2). The aetiology was always relevant, while either the SEP (model *R*^2^ = 0.353) or CMCT (model *R*^2^ = 0.409) was sufficient. Once the aetiology was considered, cranial MRI results no longer offered substantial prediction improvements in this model.

**Table 2:** Two regression models for % change in BFMDRS-m that were created in a data-driven approach. A full model containing all covariates is available in the Supplementary Material (Table S3).

	Effect	95% CI	p
*Model 1 (89 patients with CMCT measurements and BFMDRS outcomes)*			
(Intercept)	34.621	[24.126; 45.117]	<0.00001
Acquired dystonia	−29.234	[−41.794; −16.673]	0.00002
SEP abnormal	−15.231	[−27.792; −2.671]	0.01850

*Model 2 (51 patients with SEP measurements and BFMDRS outcomes)*			
(Intercept)	35.964	[27.741; 44.187]	<0.00001
Acquired dystonia	−30.513	[−40.338; −20.689]	<0.00001
CMCT abnormal	−16.163	[−29.886; −2.440]	0.02150

### 3.10 CMCT and SEP in relation to functional performance outcome (COPM-P)

One-year post-DBS COPM-P scores for children with normal SEP (*n* = 30, mean score 3.08 ± SE 0.34) were higher than those with abnormal SEP (*n* = 16, mean 1·98 ± SE 0.30) (Independent samples *t*-test: *p* = 0.020) (see Fig. S1 – Supplementary Material). However, there was no difference in COPM-P scores for children with normal versus abnormal CMCT (see Fig. S2 – Supplementary Material).

## 4 Discussion

This is the largest reported cohort of neurophysiological findings in children with dystonia and the first report to relate clinical neurophysiological parameters to outcome from DBS in dystonia. The data reflect the broad spectrum of movement disorders in children presenting to a quaternary paediatric neurology service and national neurosurgical referral centre for DBS neuromodulation therapy*.*

### 4.1 Key findings

1. Abnormal CMCT was demonstrated in 19% of children, observed across all groups, except for isolated genetic/idiopathic dystonia (Fig. 2). Dystonic disorders are not typically associated with dysfunction of the corticospinal tract. However, in many children with acquired dystonia imaging findings raise the possibility of corticospinal tract disruption. The data support previous findings that most children with dystonia or dyskinetic CP have normal CMCT even when imaging changes suggest corticospinal tract damage (McClelland et al., 2011). Many of these children had bilateral lesions on MRI and our findings concur with other reports of normal CMCT in children diagnosed with bilateral CP (Eyre et al., 2007, Park et al., 2014). It is noted that TMS activates preferentially the cortico-motoneuronal component of the corticospinal tract (Mills, 1999) and CMCT therefore only provides insight into a part of corticospinal tract function. At a pragmatic level however, demonstration of intact corticospinal projections is an important pre-surgical requirement for DBS neuromodulation (see below).
2. Approximately half the children had an abnormal cortical SEP from at least one limb. Only one child with idiopathic isolated dystonia had potentially delayed SEPs, which were probably height-related in this case. Our data are therefore consistent with adult studies reporting normal cortical SEPs (N20 response) in idiopathic/genetic isolated dystonia (Murase et al., 2000; Tinazzi et al., 2000, Frasson et al., 2001). Abnormal cortical SEPs were seen in four children with “complex idiopathic” dystonia, with unknown aetiology, no history of brain insult and normal cranial MRI, but in whom there was some additional abnormality such as mild learning difficulties. The finding of an abnormal SEP could provide further clues to the underlying pathophysiology in this group (see below)*.* The majority of abnormal cortical SEPs occurred in children with acquired dystonia, which is not surprising. Abnormal SEPs have been reported in children with ‘spastic’(Tomita et al., 2006, Riquelme et al., 2014) and athetoid CP (Tomita et al., 2006), but not specifically in dystonic CP or other forms of acquired dystonia. Indeed there is a paucity of published neurophysiological data in children with dystonia or dyskinetic cerebral palsy (McClelland, 2017). Dyskinetic CP was the largest sub-group in this study, comprising approximately 50% of our total cohort and >60% of our acquired dystonia cohort. These data therefore extend scientific knowledge in this field.
3. Neurophysiological abnormalities were present not only in children with abnormal cranial imaging, but also in those with *normal* imaging. In the SEP cohort, abnormal SEPs were identified in 8/25 children with normal cranial MRI; in the CMCT cohort, abnormal CMCTs were identified in 3/35 children with normal cranial MRI. Imaging forms an important part of the assessment of children for DBS but clinical neurophysiological parameters are often neglected. The data show that routine neurophysiological tools can provide objective evidence of sensory and motor pathway disruption even in children whose imaging is normal. This facilitates characterization of the disorder, thereby helping to manage the expectations both of the child and the family. Some children had abnormal imaging but normal neurophysiology (Fig. 2B), providing an important reminder that MRI provides a marker of underlying structure, but not necessarily function. Imaging and neurophysiology therefore both provide important and complementary information.
4. The most important clinical finding is the relationship between neurophysiological markers and outcome from DBS. Both abnormal CMCT and abnormal SEP are associated with poorer outcome, measured with BFMDRS (Fig. 4, Fig. 5). Abnormal SEPs were additionally associated with poorer outcome measured with COPM-P. A combination of both CMCT and SEP being abnormal was consistently associated with very little benefit from DBS and even a deterioration in dystonia severity, albeit with very small numbers (*N* = 4, median change −13%; range −12 to −22%). Although it is difficult to exclude all confounding factors, sub-group analyses demonstrated that the association between neurophysiology and DBS outcome was independent of dystonia aetiology and cranial MRI findings (Fig. 4, Fig. 5, Fig. 6). Multiple regression analysis also confirmed that the predictive information provided by neurophysiology findings is independent of aetiology and MRI.

### 4.2 Limitations

1. Clinical data collection was not masked to the patient’s condition. However, the SEP/CMCT findings were interpreted prior to any patient undergoing DBS and so could not have been biased with regards to outcome. The hypotheses for the retrospective review were specified prior to the analysis and this analysis was also blinded to outcome from DBS.
2. Cortical SEPs were recorded only over the standard locations for sensorimotor cortex. Re-organisation of sensory cortex could conceivably explain the inability to detect a normal cortical response in some individuals with perinatal brain lesions. However, the lower limb protocol was amended to assess a wider cortical area two years into the study period and continued to identify an increased proportion of abnormal cortical responses in the acquired dystonia group.
3. Variations in DBS electrode placement were not included in the current analysis. Previous analysis of DBS electrode placement in a group of 42 children from our cohort found no significant difference in the accuracy of electrode placement between children with primary (genetic/idiopathic) dystonia and those with secondary (acquired) dystonia (Lumsden et al., 2013a). Furthermore, Lumsden et al. (2013a) found no significant correlation between DBS outcome at 1 year and the Euclidean distance between the target and the actual electrode position. However, the possibility of some confounding effect of electrode placement on the current results cannot be excluded.
4. This is a retrospective analysis of results obtained with current clinical practice. Previous theoretical assumptions/implications regarding abnormal CMCT/SEP results will have influenced the decision of whether to proceed to DBS (see below), so the numbers of children in these groups are relatively small. Nevertheless, even with these small numbers, significant differences in outcome were seen between those with normal versus abnormal neurophysiology.
5. Outcome was assessed primarily as % or absolute improvement in BFMDRS-m score at one year. The limitations of this measure have been raised previously (Gimeno et al., 2012, Gimeno et al., 2014), particularly with regard to acquired dystonias. However, while the absolute scores may not reflect accurately the patient’s ability, the scores do correlate with other disability classification scales (Elze et al., 2016) so the differences in benefit observed between those with normal versus abnormal neurophysiological parameters are likely to be robust. In addition, COPM-P scores also differed significantly with SEP findings.
6. Longer term follow-up of the children is still required. Benefits from DBS can continue to increase for several years post-implantation, whilst for those patients with acquired degenerative dystonias, such as Neuronal degeneration with Brain Iron Accumulation or mitochondrial abnormalities, a gradual decline in DBS efficacy is often seen. An on-going longer-term follow up study for these (*n* = 12) and indeed all the children who proceeded to DBS is therefore appropriate.

### 4.3 Interpretation

Whilst it is not unexpected that a proportion of children in this cohort would show neurophysiological abnormalities, the prevalence of sensory pathway abnormalities is striking. The finding of greater sensory than motor pathway disruption in the present cohort is consistent with diffusion tensor imaging studies that revealed a higher proportion of abnormalities in thalamo-cortical sensory than motor pathways in children with ‘spastic’ CP and periventricular leukomalacia (Hoon et al., 2002, Hoon et al., 2009). In our cohort, the imaging finding most commonly associated with abnormal SEPs was evidence of periventricular white matter damage, therefore extending these observations to children with predominantly dyskinetic (rather than spastic) CP.

Whilst it is not possible to conclude from these data that the sensory abnormalities are causal, it is likely that disruption of sensory pathways contributes to the disordered motor control in these children. Several studies have demonstrated anomalous sensory processing in children with “spastic” CP (Riquelme and Montoya, 2010, Kurz et al., 2014, Kurz et al., 2015a, Riquelme et al., 2014) and these abnormalities do appear to have functional importance (Kurz et al., 2015b). There is clear evidence that primary dystonia is a network disorder involving multiple brain regions, including the cerebellum and sensorimotor cortex (Neychev et al., 2011, Neumann et al., 2015) and is a disorder of sensorimotor integration (Hallett, 1995, Tinazzi et al., 2000, Abbruzzese et al., 2001, Frasson et al., 2001, Abbruzzese and Berardelli, 2003; Tinazzi et al., 2003). Reduced pre-movement gating of incoming sensory information has been demonstrated in adults with writer’s cramp (Murase et al., 2000), and abnormal cortical plasticity in response to afferent stimulation has been demonstrated in adults with idiopathic/genetic isolated dystonia (Quartarone et al., 2008, Tamura et al., 2009). These abnormalities may lead to sensory information being distorted, resulting in abnormal motor output (Frasson et al., 2001, Tinazzi et al., 2003). Although the sensory pathway abnormalities in idiopathic/genetic dystonia occur at cortical processing level (e.g. reduced inhibition of afferent inputs) (Hallett, 2011) we postulate that in children with dystonia arising from other causes, disruption to the sensory system at other nodes of the network may well contribute to the disorder. For example disruption of somatosensory pathways arising from perinatal brain injury may also lead to abnormal perception of sensory information, which in turn leads to a distorted motor output. Indeed, if these abnormalities have been present since early life, including within ‘critical’ and ‘sensitive’ windows for development (Ismail et al., 2017), they may disrupt the maturation of normal sensorimotor networks and the development of normal motor programmes may never occur. The differential outcome for acquired versus isolated idiopathic/genetic dystonia certainly suggests that the underlying pathophysiological mechanisms of the movement disorder are likely to be different (Neychev et al., 2011) and several recent studies have provided evidence to this effect (Trompetto et al., 2012, Kojovic et al., 2013, McClelland et al., 2016).

The findings in children with complex idiopathic/genetic dystonia are worthy of particular comment. These are children with unknown aetiology, no history of brain insult and normal cranial MRI, but in whom there was some additional abnormality such as mild learning difficulties. Previous studies of SEPs in dystonia have tended to exclude such patients and have focused on isolated idiopathic/genetic dystonia. We included these children in our cohort as they represent an important part of our caseload and, as with the acquired dystonia patients, outcomes from GPi-DBS are more variable and harder to predict. Within this complex idiopathic /genetic dystonia group, three children had abnormal CMCT and four had abnormal SEP. The finding of an abnormal CMCT or SEP could provide further clues to the underlying pathophysiology in this group and we consider that these findings demonstrate the additional value of neurophysiology in their assessment, since their cranial imaging was normal.

The relationship of neurophysiological parameters to outcome has important clinical implications and also promotes interesting scientific discussion regarding the underlying mechanisms. Theoretically a functioning CST is required for DBS to be effective, as, regardless of how pallidal DBS modulates brain network activity, an intact motor outflow pathway is needed. The current data support this concept: no children with absent MEPs went forward for DBS, but a small number of children with prolonged CMCT (indicating some dysfunction of the CST) did receive DBS and the results indicate that these children are less likely to benefit from DBS. Clinical spasticity is associated with less benefit from DBS (Romito et al., 2015) but CMCT can provide additional information about corticospinal tract function where examination findings do not reveal clear spasticity.

The reason for an association between abnormal SEPs and response to DBS is not clear. The mechanisms by which DBS improves motor control are not yet understood, but it is becoming evident that stimulation of the GPi leads to widespread brain network changes: in idiopathic/genetic isolated dystonia DBS reduces cortical plasticity (Tisch et al., 2007) and attenuates pathological low frequency oscillations between the basal ganglia and sensorimotor cortex (Barow et al., 2014). Modulation of sensorimotor cortex by DBS has also been demonstrated with Deep Brain Stimulator Evoked Potentials (DBSEPs) (Bhanpuri et al., 2014), likely to be mediated via pallido-thalamo-cortical projections (Tisch et al., 2008). The importance of these projections is implied from the finding that the highest amplitude DBSEPs are evoked from those electrode contacts found clinically to produce the most benefit (Tisch et al., 2008, Bhanpuri et al., 2014), providing further evidence that modulation of the sensory network is relevant both to the mechanisms underlying dystonia and to the therapeutic effects of DBS. Our findings indicate that in some children with dystonia secondary to perinatal brain injury, there is abnormal development of the sensorimotor network as measured with SEPs. This could reflect dysfunction at the cortical level, thalamic level, thalamo-cortical pathways or a combination of these. If the network is mal-developed at the level of the sensorimotor cortex, then part of the very substrate that DBS is modulating may not be able to respond, leading to less benefit. If the abnormal SEP reflects thalamic or thalamo-cortical pathway dysfunction then this in turn may impede the transmission of the effects of pallidal neuromodulation via the pallido-thalamo-cortical pathway to the sensorimotor cortex, therefore “blocking” any effective improvement in motor control. Alternatively the abnormal SEPs may simply indicate a greater severity of overall brain network disruption. Elucidation of the precise mechanisms requires further studies, but meanwhile, the present findings have potential clinical utility in guiding selection/stratification of children for DBS and counselling of families. This is particularly relevant in children with acquired or complex idiopathic dystonia, in whom outcomes vary widely between individuals (Vidailhet et al., 2009, Koy et al., 2013, Olaya et al., 2013).

### 4.4 Relevance to clinical practice

The service model adopted by our clinical service is described in Gimeno and Lin (2017). Clinical factors which tend to lead to the child being considered unsuitable for DBS include fixed contractures or a motor phenotype dominated by spasticity. In practical terms, up until this analysis, the finding of isolated CMCT or SEP abnormalities involving one or two limbs resulted in the communication to the parents and carers that DBS was unlikely to provide relief of symptoms in those limbs, whereas there remained the possibility of DBS efficacy in those limbs with normal neurophysiology findings. Conversely, in the context of normal CMCT and SEP results we have tended to be more optimistic about the potential outcome of DBS as this confirmed that these pathways were physiologically functional. This approach was based upon theoretical arguments as outlined above, as there are no previously published data on this topic. Overall, few patients with abnormal CMCT proceeded to DBS and those that did all had prolonged CMCT, rather than absent MEPs. No patients with abnormal CMCT and SEP to all limbs went forward for DBS. In addition to the neurophysiology findings, there were multiple other reasons why a child may not have proceeded to DBS, including findings from the multi-disciplinary clinical assessment and family preference, fears and expectations, even in “ideal” cases.

The numbers of patients with abnormal CMCT/SEP and DBS outcome data are therefore rather small, and this necessitates caution in interpreting the results. Validation by further studies is desirable. Nevertheless, the outcomes for children with abnormal CMCT or SEP were consistently poor with median% change in BFMDRS-m score in these groups being -4% and -8% respectively (Fig. 4, Fig. 5). Moreover, no patients in the abnormal CMCT or SEP groups showed an improvement of more than 25% in BFMDRS-m. The finding that neurophysiological evidence of abnormal motor or sensory pathway function is associated with less good outcome from DBS is consistent with both theoretical arguments and clinical findings (Romito et al., 2015). In addition, the consistency of the relationship for SEPs across two different outcome measures (one impairment based and the other function/goal-based) is notable. Overall, we interpret these results as suggesting that abnormal neurophysiological parameters should be considered a *relative* contra-indication to DBS.

Although the presence of normal physiology appears to confer some advantage to the child potentially undergoing DBS for dystonia, there remains considerable variation in response even within this group and so the precise amount of benefit cannot yet be predicted. Indeed, when considered in terms of sensitivity and specificity, the *specificity* of an abnormal SEP and/or CMCT for detecting patients who will have a poor response to DBS appears to be considerably stronger than the *sensitivity* of the tests for identifying such patients (see Fig. 4, Fig. 5 and Supplementary Material). Further predictive markers are therefore required.

The observation that a combination of abnormal CMCT and SEP was associated with an apparent deterioration in dystonia severity following DBS is of high importance. The numbers are small (only four patients in this category), so again the results need cautious interpretation and ideally validation in future studies. However, the specificity of a combined abnormal CMCT and SEP for a poor response to DBS was high and this effect was observed regardless of aetiological group or imaging findings. Particular caution may therefore be warranted in counselling these families about the potential role of DBS and consideration should be given to alternative therapies/ management strategies. Some benefits reported by families, such as improved comfort or pain control are not captured by standard assessment scales and one test alone does not necessarily rule out DBS for an individual patient. Each child undergoes a detailed multi-modal and multi-disciplinary assessment. However, the current findings suggest that neurophysiological markers make an important contribution to this assessment and can inform the stratification of children and counselling of families for precision DBS surgery. Valuable additional information is gained, which is not available through imaging alone. Where one modality is unavailable (e.g. due to contraindication to TMS) the CMCT or SEP findings alone each appear to have some clinical utility in helping to stratify patients for DBS. The role of SEPs appears particularly important since these results correlated not only with change in dystonia severity (BFMDRS-m), but also with achievement of functional goals (COPM-p). However, it is preferable that both CMCT and SEP studies be performed for all limbs since the combined CMCT/SEP results were more informative than either of the individual tests in isolation.

## 5 Conclusion

The current findings address the paucity of published neurophysiological data in children with dystonia and provide insight into the expanding spectrum of individuals with complex movement disorders being referred for DBS. Referrals for individuals with acquired dystonias are increasing. Outcomes in this group are more variable and a clear strategy is required for selecting children for neuromodulation and managing patient/family expectations regarding the potential benefit. This is a single, retrospective study with small numbers in some sub-groups, which should be taken into account when interpreting the findings. However, the apparent disadvantage to those with abnormal CMCT and SEP is striking. We consider that the present data demonstrate the value of CMCT and SEPs in the selection process and would therefore recommend that these neurophysiological parameters be included alongside multi-disciplinary clinical, therapy and imaging assessments of potential candidates for DBS. Further predictors are still required, emphasizing the need for continued multi-modal data collection and follow-up in order to arrive at the ideal of offering a personalized prognosis for each child referred for possible DBS neuromodulation for dystonia.
