# Supplementary material for: Somatosensory Evoked Potentials and Central Motor Conduction Times in children with dystonia and their correlation with outcomes from Deep Brain Stimulation of the Globus pallidus internus
Source: Clin Neurophysiol. 2018 Feb;129(2):473–86. doi: 10.1016/j.clinph.2017.11.017 (PMC5786451; doi:10.1016/j.clinph.2017.11.017)
Supplement: Supplementary data 1 [file mmc1.docx]

**Somatosensory Evoked Potentials and Central Motor Conduction Times correlate with Deep Brain Stimulation outcomes in Childhood Dystonia**

Verity M. McClelland, Doreen Fialho, Denise Flexney-Briscoe, Graham.E. Holder, Markus C. Elze, Hortensia Gimeno, Ata Siddiqui, Kerry Mills, Richard Selway, Jean-Pierre Lin

**Appendix of Supplementary information**

|  | **Normal** | | | | **Abnormal** | | | |  |
| --- | --- | --- | --- | --- | --- | --- | --- | --- | --- |
|  | **Outcome: % improvement in BFMDRS-m score** | | | | **Outcome: % improvement in BFMDRS-m score** | | | |  |
|  | n | Mean | Median | Range | n | Mean | Median | Range | **p value** |
| **CMCT** | 78 | 15.23 | 7.44 | -31.1 to 88.0 | 11 | -5.26 | -4.12 | -21.9 to 14.0 | **0.002** |
| **SEP** | 35 | 16.25 | 5.88 | -10.1 to 79.2 | 16 | -4.36 | -7.72 | -21.9 to 22.9 | **0.001** |

**Table S1.** Comparison of DBS outcomes in children with normal versus abnormal CMCT and normal versus abnormal SEP. The p-values are calculated using Mann-Whitney tests.

|  |  | **Outcome: % improvement in BFMDRS-m score** | | |
| --- | --- | --- | --- | --- |
| **Combined Neurophysiology Result** | n | Mean | Median | Range |
| **Both CMCT and SEP normal** | 25 | 19.65 | 7.69 | -10.1 to 79.2 |
| **Either CMCT or SEP abnormal** | 11 | 2.28 | 1.38 | -10.1 to 22.9 |
| **Both CMCT and SEP abnormal** | 4 | -15.15 | -13.32 | -21.9 to -12.1 |

**Table S2.** Comparison of DBS outcomes in relation to combined Neurophysiology results.

**Sensitivity and Specificity of abnormal neurophysiological parameters for predicting poor outcome**

Although some of the sub-group numbers in this study are small, it can be helpful to consider the findings of abnormal CMCT/SEP in relation to DBS outcome in terms of their sensitivity and specificity.

For example, the sensitivity and specificity of CMCT or SEP alone or combined as a predictor of “null response” to DBS are as follows (using an arbitrary cut-off of 0% change in BFMDRS-m score):

CMCT alone:

Sensitivity for predicting a null response = 25%

Specificity for predicting a null response = 94.7%

Positive predictive value of abnormal CMCT predicting a null response = 72.7%

SEP alone:

Sensitivity for predicting a null response = 46.1%

Specificity for predicting a null response = 84%

Positive predictive value of abnormal SEP predicting a null response = 75.0%

Combined CMCT/SEP:

Sensitivity of both results being abnormal for predicting a null response = 21%

Specificity of both results being abnormal for predicting a null response = 100%

Positive Predictive Value of both SEP and CMCT being abnormal predicting null response = 100%

**Central Sensory Conduction Times**

Where possible the Central Sensory Conduction Time was calculated and recorded in the clinical report. However, due to the severity of the movement disorder in many children, the cervical and lumbar traces were contaminated by muscle and movement artefact, which precluded accurate quantification of these components, in turn precluding calculation of the Central Sensory Conduction Time. Central Sensory Conduction Times (CSCT) were available for 142 limbs in 67 patients. In contrast, the cortical components were a more robust measure, with satisfactory cortical traces obtained in the majority of cases (368 limbs in 100 patients). CSCT was prolonged in six patients in whom the latency of the first cortical component was also prolonged. There were no patients in whom the cortical SEP latency was normal but central SCT was prolonged. There was one patient in whom the cortical SEP latency was mildly prolonged from both upper limbs but in whom the central SCT was within normal limits. This child has recently proceeded to have DBS.

**Multiple Regression analysis**

As an additional sensitivity analysis for confounding, linear regression models were fitted in a data-driven approach. Variable selection for the models was done using a stepwise procedure that successively added / removed variables one at a time to find the best fitting model. Likelihood-ratio tests were used to compare models. In a forward selection approach, the variables that most improved the model were successively added. Once no variables significantly improved the model anymore, a backward elimination approach successively removed variables that no longer significantly improved the model. This was followed by another round of forward selection and backwards elimination. If an interaction term was included, both underlying variables were automatically also included in the model.

|  | **Estimate** | **95% CI** | **p** |
| --- | --- | --- | --- |
| (Intercept) | 36.073 | [22.961; 49.185] | <0.00001 |
| Acquired dystonia | -30.897 | [-42.702; 7.966] | 0.00951 |
| CMCT abnormal | -17.368 | [-35.767; 2.563] | 0.17260 |
| SEP abnormal | -16.602 | [-53.742; -8.053] | 0.08733 |
| Both CMCT and SEP abnormal (interaction effect) | 10.804 | [-27.927; 49.534] | 0.57452 |
| MRI abnormal | 3.786 | [-18.846; 26.418] | 0.73598 |

**Table S3.** Full regression model for % change in BFMDRS-m for the 40 patients for whom outcome, imaging, CMCT and SEP data were available. The relatively low number of patients for whom all measurements are available is one of the reasons why p-values are worse compared to the selected models (Table 2). With R²= 0.425 this full model does not offer a substantial improvement over the selected models (presented in the main manuscript).

**Figures showing analysis of COPM-P data.**


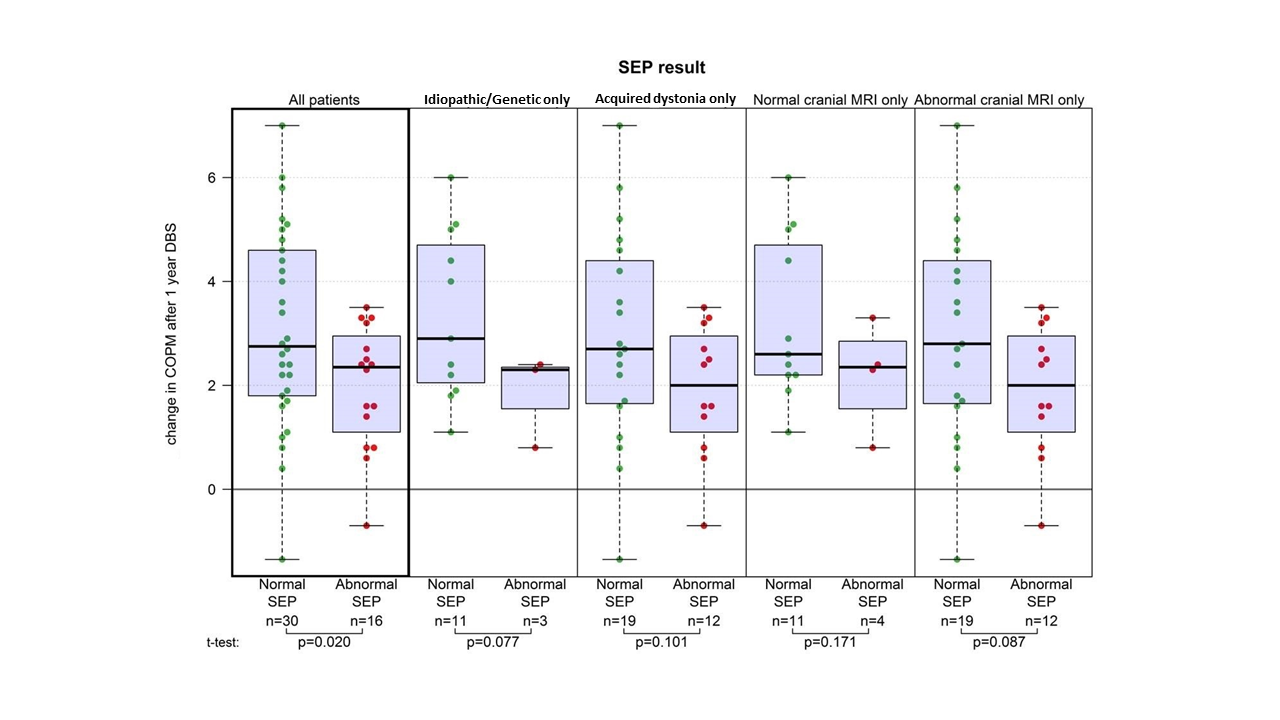


**Figure S1.** Box and Whisker plots showing outcome from DBS measured as change in COPM-P score at 1 year after surgery in relation to SEP result. The p-values are calculated using independent samples t-tests.


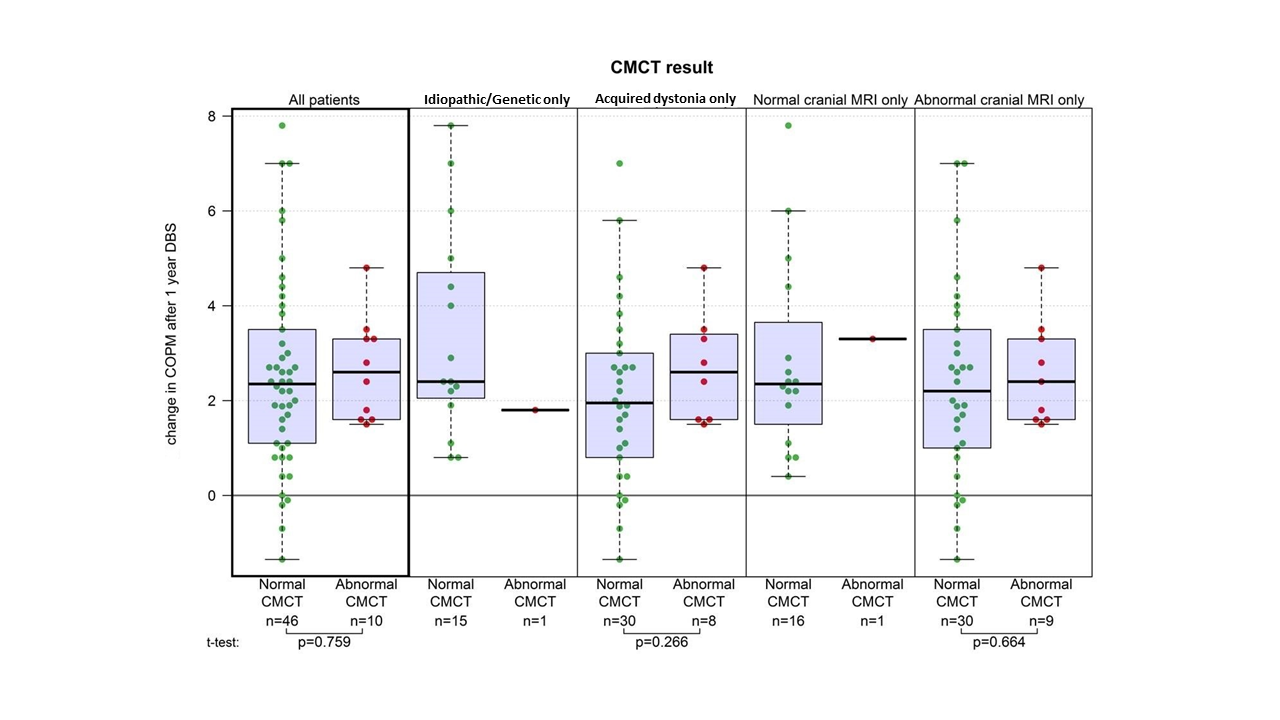


**Figure S2.** Box and Whisker plots showing outcome from DBS measured as change in COPM-P score at 1 year after surgery in relation to CMCT result. The p-values are calculated using independent samples t-tests.
